# Proceedings of the Society for the Study of Comparative Psychology in Connection with the Faculty of Comparative Medicine and Veterinary Science of McGill University, Montreal, Canada

**Published:** 1894-12

**Authors:** 


### PROCEEDINGS OF THE SOCIETY FOR THE STUDY OF COMPARATIVE PSYCHOLOGY IN CONNECTION WITH THE FACULTY OF COMPARATIVE MEDICINE AND VETERINARY SCIENCE OF MCGILL UNIVERSITY, MONTREAL, CANADA.

To economize space, the proceedings of the meetings are given in order, without further preface. They were all held in the lecture room of the Faculty, in the evening, and the President, Prof. Wesley Mills, occupied the chair at every meeting.

I.

The annual opening meeting of the Society for the Study of Comparative Psychology was recently held in the lecture room of the Faculty of Comparative

Medicine of McGill University. Dr. Wesley Mills, through whose influence the society was founded eight years ago, presided. The attendance was good and a large number of new members were proposed. The financial report also showed general prosperity, considerable money being voted for the purchase of books for the small but excellent library of the society.

The usual opening address was on this occasion unavoidably dispensed with, and, in lieu thereof, the society listened to the reading of a paper written by Prof. J. E. Le Rossignol, of the University of Ohio, entitled "Malevolence in the Lower Animals." At the outset the writer declares the limits of study in comparative psychology to be fixed. Only those psychical states of the lower animals are intelligible to us which are similar to our own. That which we do not experience or possess in some measure we cannot conceive in the lower animals. In the ravenous feeding of a dog after a prolonged hunt, we behold a feeling of hunger similar to that in our own experience. If reasoning from human analogy, we credit the animal with feelings of hunger, thirst and pain, so we can find a basis for similar states of joy, grief and anger, in fact, all the chief sensations and emotions that regulate the life of man. In the light of evolution the probability of such analogy is much increased. It is evident that the emotions must be studied by their psychical expression. No one can fail to see the essential resemblance between an enraged animal and a furious man ; and when we consider that the exciting cause is the same in both cases, the presence of a similar emotion is most reasonable. In most cases anger is connected with attack or defence. The heredity of anger, a subject of importance to the breeder, is most evident in the domesticated animal, whose character has been profoundly altered by association with man. Hereditary malevolence may be said to exist in a latent form, consisting in a certain irritability of the nerves, the capacity for anger rather than anger itself being inherited. The acquired impulse is exemplified by every ill-treated animal. The dreaded "rogue elephant," ostracized by his companions, develops unusually bad temper.

II.

The following questions bearing upon the heredity of bad temper in dogs had been submitted to Dr. Wesley Mills and others :

1. Is bad temper hereditary ?

2. Is training or environment of greater importance than heredity in determining the good and bad temper of a dog ?

3. Are good and bad temper respectively characteristic of any particular breed of dogs ?

4. What is the proper method to employ in overcoming hereditary bad temper in a puppy ?

5. What sort of treatment is calculated to spoil the temper of a puppy ?

Dr. Mills answered as follows :

1. I am satisfied that any trait may be hereditary, bad and good temper included. I would hesitate to breed from a bad tempered dog of either sex, no matter how excellent a specimen otherwise, especially if of a larger breed, as being more dangerous to man and his canine companions.

2. Heredity I believe by far the stronger.

3. No ; though it occurs more frequently among certain breeds, perhaps. A bad tempered setter or spaniel is rare. But bad temper may be said to be much more common in certain breeds than in others, rare in some, but absolutely confined to none.

4. Furnish an environment free from temptation and encourage a sense of justice—for dogs have this in a fashion at all events—from the first. Give him little chance to quarrel, but insist on his realizing that if he does it willingly, punishment of some kind, not necessarily corporal, will follow. I have found separation sometimes a severe punishment for dogs, they seem to know in some measure what it means. Disapprobation, scolding, is severe punishment for some dogs.

5. Teasing, hard usage, over punishment, injustice, etc. Highly bred dogs especially, suffer much physically under bad usage, especially punishment in anger and to excess.

The chief causes or occasions of malevolence are those connected with obtaining food, and those connected with reproduction. Only the fittest will survive the battles resulting from such malevolence, and thus does the latter tend toward the preservation of the species.

It may be gathered from the above that the methods for the suppression of malevolence should have a scientific basis. Exclude the causes. Intelligent regulation of breeding, the exclusion of corporal punishment, the development of affection, the constant companionship of a refined man—in whom the moral sense will dominate the malevolent emotions and prevent their imitation—will all aid in the suppression of malevolence.

So many of these suggestions are applicable to the infant that it recalls the words of Dr. Mills, who asserts: "It is certain that the dog may be treated in all respects more as if he were a child than as bearing any close relationship to our other domestic animals."

After some criticism on the paper the meeting adjourned.

III.

Messrs. Thomas and Carey contributed papers, Mr. Thomas reading one entitled "Thoughts on the Psychic Processes in Lower Animals," in which he discussed several facts which had come under his direct notice, among others a very interesting case of a pointer dog belonging to a family residing in the city. The family always spent their summer vacation about one hundred and twenty miles back in the country, and the dog being a favorite animal always accompanied them, riding in the same car. Last fall when the family was returning home, accompanied, as usual, by the dog, the conductor coming into the car objected to the animal riding with the passengers and removed him to the baggage car, where he was tied up. However, this must have been done insecurely, as the dog got loose and jumped from the train when about sixty miles from home. The family was immediately notified and the railway officials sent telegrams to all their agents along the route, but their efforts were unavailing, as the dog had disappeared. About a week after the above event the mistress of the dog, who had been granted a pass, good for one month, took a trip along the line in search of the missing animal, and, when on the way back at a station twenty miles from her own town, to her intense surprise the dog came into the car she occupied and walked up to her. How can we account for this strange event? Was it by sense of smell or do these animals possess faculties not possessed by men?

Mr. Carey followed with a paper entitled "Psychology of the Honey Bee," in which he narrated some very interesting and peculiar facts in regard to the habits of these insects.

After listening to and discussing the above papers, Mr. Hall related a case deserving of more than passing notice. Some men were engaged in blasting a short distance from this city. Their foreman was in the habit of calling out "Helloa!"

before firing the fuse. A flock of chickens in the vicinity had learned to recognize danger when they heard this signal, and always started off for a barn near at hand. On Mr. Hall going over and questioning the men in regard to the matter, he was informed that when the first two or three blasts had been set off, no attention whatever was taken of them by the chickens, and the effect had been somewhat disastrous to their flock, but after that, evidently recognizing danger, they always sought their place of shelter. In order to see what the effect would be were he to give the signal, Mr. Hall called out "Helloa!" but no notice whatever was taken of his cry, but on the foreman calling out again they set off once more. How can we account for these strange actions?

IV.

Mr. Salley read a paper entitled "Do the Dog and Horse Possess all the Faculties of Man?" in which he reasoned that as both man and beast possessed brains, and a nervous system of similar construction, it was natural to conclude that both had reasoning power, and if granted this, then, do they not possess all the faculties of man?

This called forth discussion, and the questions considered in connection with Mr. Salley's paper were: "Have the lower animals rights in the strict sense of the term?" "Can lower animals be taught to articulate?" "If animals possess no reasoning power, have they no rights?" And lastly, "Have we any right to deprive animals of their liberty?" In regard to the latter question Dr. Mills expressed himself most emphatically as follows: "We have no right to interfere with the pleasures of the lower animals, except to advance the welfare of all sentient beings, i. e., of the animal creation as a whole."

Mr. Shaw followed Mr. Salley, reading a paper entitled "Animal Psychology," in which he dwelt upon the reasoning powers displayed by the dog, contending he was influenced by relationship to man, for centuries having been his companion and adapting himself to his many wants and even caprices. Mr. Solandt then occupied the attention of the meeting while he read "Animal Psychology as Viewed by a Student of Comparative Medicine." He also claimed that animals really possess reasoning power, and proceeded to show the difficulties they contended with owing to the shortness of their lives, lack of instruction, etc. He also related several interesting occurrences in regard to animals, concluding an eloquent paper by stating that he thought only those who were willing to look after and care for dumb creatures should be allowed to have possession of them.

V.

A regular meeting of the Association for the Study of Comparative Psychology was held in the lecture room of the Faculty of Comparative Medicine and Veterinary Science, the president, Prof. Wesley Mills, in the chair.

The first essay of the evening was read by Mr. Hollingsworth, entitled "The Reasoning powers of Animals." The writer first points out the difficulty of the subject, inasmuch as the term psychology does not admit of an easy definition or one which is clearly comprehensible. He illustrates what is generally understood by the term psychology or the science of mind by stating that two different principles make up life, as primary and secondary, one the originator and the other executor of all things, mind and body being associated. A man walking towards the north end of the city suddenly stops, turns around and walks south, the mind compelling the body to perform this movement. He then shows the analogy which exists between man and the lower animals by the latter possessing a mind, though of an inferior order. Memory is a faculty or quality developed to a high degree in them, as will be seen in horses and dogs, and illustrated by personal observation of the writer, as in the case of a dog running at full speed, and seen to stop, turn around and run in the opposite direction, owing to sudden recollection of something; a horse pulling the peg out of a gate so as to let himself into a rich pasture, because he had seen his master do the same many times.

Mr. Buchan presented an elaborate paper on "The Psychic Development of the Lower Animals Compared with that of Man." The writer defines psychology as the science which treats of the facts and phenomena of self. The main characteristics of self are consciousness, which not only exists, but is cognizant of the fact. In all animal life there is a feeling of separate existence, but not of equal intensity. By descending the animal scale, features and characteristics are lost, although some of the lower animals exhibit a high development in the special senses and the muscular system, but there is a gradual descent until we get to the lower forms where mental faculties are not present or special senses developed to any extent; but still there is an existence which can be imagined as a hazy state of consciousness composed of a blending of the sensations which develop distinctly in higher animals. Man is considered to be possessed of higher mental qualifications than any other animal in the scale, which must be brought about by development. At birth he is inferior in many respects mentally and physically. The growth of psychic power in the human subject gives rise to three questions: 1. What element enters into this development? 2. How is this element introduced? 3. Why does each particular mind develop in its own peculiar manner? The writer states that development of mind is due to certain factors whose existence, properties and combinations we seek in psychology. Education is introduced and accomplished by the will, the action of which is not always so apparent as in the case of the senses. Information being received may produce a change in the mind, as joy, sorrow, hatred, love, satisfaction, etc. The peculiar manner in which each mind develops is owing to the fact that man inherits a great variety of feelings, appetites, passions, and makes effort to obtain knowledge and perform actions to gratify feelings. By the psychic powers in man comparison with the lower animals are considered. The elements making up their psychic development are the same as the man, the only difference being in degree rather than kind. Their experiences are different, being due to difference in environment. By observation the writer states that lower animals hold communication with each other, and as their needs are few compared with man's, their vocabulary is sufficient. There is no doubt regarding communication between animals of the same kind. Education in the lower animals is seen apparently in the case of a robin It built its nest where its movements could be watched. About one week before the young birds were ready to leave the nest, the mother bird gathered material and built an entire new border around it, the young watching the performance. This looked like giving the young birds a lesson in practical work. Animals learn but very little by observation from other species. As evidence, take a horse, say, thirty years old. The greater part of that time he may be tied to a ring in his stall. Every day his attendant will untie him and take him out; still he will stand in that position before he will attempt to untie his rope. The reason seems to be the inability to connect this action of man with one he might perform himself. Many horses have learned to do this, not by observation, but by experience. Man's association with the lower animals develops them to a high degree, due to changes in environment and experiences, which is a modified source of information. Evidence of animals' feelings can be plainly seen in their expressions of joy, sorrow, etc., also in receiving their first lessons in training. Experience teaches him that resistance is useless. The writer states that the maximum of success can be reached by kindness and firmness in the education of lower animals. Injudicious force and cruelty will develop hatred and distrust, and animals may seek opportunity for revenge. Occasionally we meet with animals possessing almost human intelligence; but the average animal possesses these faculties to a certain degree only.

Mr. Hall read an interesting paper on "Animals possess reasoning power, but not to the same extent as man." Language in animals is stated by the writer to exist, and animals try to learn man's language to a greater extent than man tries to learn theirs. He also compared the language of lower animals with that of some of our Indian tribes, whose language is composed only of seventy-three words. Instinct is treated under the heads of organization, heredity, education, imitation. The author thinks nature has so related all faculties that if one is lost it is made up by more special development of some other, as loss of sight may render the sense of touch more acute. Language does not come natural to a child although it is inherited to a certain extent. Imitation plays an important part, for a child will learn a foreign language. Birds have been found singing differently from the style characteristic of their species. Instincts are crystallized habits, comprising those faculties of mind which lead to conscious performance of action that are adaptive in character and showing the knowledge of relation to the means employed and the ends attained. Domestic instincts being less fixed than natural, the writer makes a comparison of the brains of different groups of animals, stating the same chain of connection exists, though differing in size, in the different animals and in the same parts. Many animals during infancy surpass the child in intelligence, and experience in old age that stage of dotage similarly to man. That dogs have a sense of cleanliness is clear, but that they have a sense of right and wrong is an open question.

VI.

Messrs. McAlpine, Morin and McLeod contributed papers. Mr. McAlpine's subject was "Thoughts on Psychology," in which he proceeded to argue that there was an exchange of ideas among the lower animals, and held that no reason existed why man could not learn their language if he only devoted sufficient time and labor to accomplish this end. A great many difficulties would certainly have to be overcome, but the end, if accomplished, would be as great a boon to mankind as many discoveries which have taken centuries to complete. Mr. McAlpine related an incident that had come under his direct notice in proof of his statements. A railway was built through a pasture where some cattle were kept, and on the first train passing through they exhibited great fear and ran to the furthest end of the field. This they continued to do for some time, but by degrees they became used to the new sight and would graze near by without taking any notice of the cars. Later on in the season other cattle that had never seen a train before were placed in the same field, and when the first train passed they exhibited great fear and would doubtless have acted as the others had formerly done had it not been for their example. They stopped grazing and, running towards them, seemed by a series of motions and expressions to make them understand there was no danger, and although the strange animals at first exhibited a little distrust, they returned and never afterwards showed any signs of fear. The essayist then proceeded to point out the intelligence exhibited by some animals in one way or another—in some cases to an extraordinary degree in one particular direction, thus proving that various talents were present and only lie dormant for want of a stimulus, and if proper means were taken they might possibly be all developed in one animal.

Mr. Morin then proceeded to read a paper on "Instinct by Natural Selection," in which he alluded to the migratory instinct in birds, the wonderful abilities displayed by some young broods which fly by themselves and are not accompanied by older birds, flying over enormous tracts of land and sea without guidance or previous experience. Also related to these birds are others, such as carrier pigeons, which, although not migratory, have the "homing" faculty so well developed Again, he alluded to the propensities of young pointers and setters to point the first time they, are taken out, retrievers to retrieve, and to young sheep dogs having the tendency to run around a flock of sheep instead of at them, as many other dogs would do. These latter facts are all alluded to by Darwin, whose accuracy on such a subject is not likely to be disputed. Darwin also has pointed out a serious difficulty in his theory of the origin of instinct by natural selection. It is that among various species of social insects, such as bees and ants, there occur neuter or asexual individuals, who manifest entirely different instincts from the other or sexual individuals, and, as the neutrals cannot breed, it is difficult to understand how their peculiar and distinctive instincts can be formed by natural selection, which requires for its operation the transmission of mental faculties by heredity. Darwin supplies the only possible means by which this problem may be solved, viz., "by supposing that selection may be applied to the family as to the individual."

Mr. McLeod then proceeded to read his paper, "Remarks on the Psychic Capabilities of Animals," and confined his remarks principally to the dog, commenting on the talents and sagacity displayed by this animal, and claimed that when improved by association with man, they became, to a certain extent, rational beings. His courage is unbounded, he appears never to forget a kindness, but soon loses recollection of an injury if received from his master or from one he loves. His docility and mental pliability are great, his habits social, and his fidelity not to be shaken ; his discrimination is equal in many respects to human intelligence, and if he commits a fault he is sensible of it, and shows pleasure when commended. Mr. McLeod alluded to the dog when in a wild state only howling, but on becoming the friend and companion of man he has then wants and wishes, joys and fears, to which in his wilder state he appears to have been a stranger ; his vocabulary, if it may be so called, increases and he is able to express his enlarged and varying emotions. He anticipates rewards and punishments, learns to solicit the former and depreciate the latter, evidently discerns days of household mirth or grief, and deports himself accordingly. Let us look at the power of calculation in animals ; that they possess a wonderfully correct knowledge of time and its flight cannot be doubted, but what the nature of this knowledge is and how acquired is not so apparent. Many dogs, cats, fowls and even wild birds know almost within a few minutes man's meal hours. Milk cows know when it is their time to be milked and fed, as anyone who chooses to observe these animals will perceive. Several other points were made by the essayist before concluding.

The president, Dr. Mills, complimented the three gentlemen on the evident care and thought shown in their papers.

VII.

The following gentlemen contributed papers: Mr. Moore, "Intelligence of the Lower Animals," and Mr. Mulvey, "Psychology of Man and the Lower Animals Briefly Compared." Mr. Moore, in his paper, related and discussed some interesting experiences bearing on reasoning power in lower animals, the following among others : A horse had been in the habit of taking the children to school in the morning, being left to return home by himself. One day, instead of going home, he went to the blacksmith's shop, which was quite out of his usual route. The smith, thinking the animal had wandered from home, tried to send him away, but the horse persisted in remaining. Finally, he held up one foot, and upon examination a stone was discovered wedged in between the frog and the shoe. The stone was removed and the animal went straight home. Surely we have reasoning powers in this case.

Mr. Moore also related a circumstance which goes to prove that the lower animals have retentive memories. An elephant which was kept for the purpose of assisting in loading and unloading lumber from boats was given a piece of tobacco by one of the men working near. Thinking it was candy, he put it into his mouth, but as soon as he tasted it, he made a rush for the fellow, who, however, managed to elude him and made his escape. Next season, which was several months after this incident, the same man appeared on the scene and was not paying any attention whatever to the elephant when the latter suddenly began to make towards him, and it was only with difficulty that the man made his escape.

Mr. Mulvey followed with his paper, showing that while the lower animals were lacking in regard to development of some functions, when compared with man, yet we must acknowledge they possess others more acute.

The president complimented the writers on the care evinced in the preparation of two such papers of the merit which the above account gives a very inadequate conception. Mr. Moore's paper showed a praiseworthy amount of research, and Mr. Mulvey's observations turned to good account. The discussions hinged largely on the question : Can animals reflect in the proper sense of the term?

### VIII.

The essayists of the evening were Messrs. Anderson, Baker and the secretary-treasurer, Mr. McGillivray. The first paper read was by Mr. Anderson, who discussed "Practical Thoughts and Facts in Connection with Comparative Psychology," contending in his paper that psychology was not merely a word in composition, but a study in which the use of science, common sense, time and thought would not be misapplied. Instances of memory are exhibited in the dog just as acute as in man, in fact anatomically, physically and psychologically to a certain degree we are closely allied to the lower animals. Man is blessed with talents many of which are refined in nature, likewise the dog; for example, music is claimed to be refining, and without doubt is. Who has not seen a dog sitting for a length of time listening to piano music, and if one were to produce a marked discord the animal would immediately express his feelings by a howl. Mr. Anderson then gave some of his personal experiences in connection with the lower animals, among others a rather interesting one concerning a dog in his possession. The dog had conceived a great liking for one of the members of the household, and nothing pleased him better than being allowed to rest at the room door. The room in question was situated at the end of a hallway, which was covered by oilcloth, consequently any person immediately below could hear the dog when his claws came in contact with the glazed surface. One day the dog as usual had taken up his abode at the bedroom door, but was sent down-stairs ; however, in a little while he was heard again crossing the oilcloth up-stairs. Before removing he was this time given a good scolding, and it was easily seen from his looks that he was ashamed of himself. One of the members of the household then went into a room opposite the before-mentioned one, and, leaving the door slightly open, awaited further developments. In a short time the dog was seen approaching, walking with a peculiar gait ; the whole weight of his body was placed on the ball of his foot, and it was impossible to hear his claws touch the oilcloth. Have we not got here a perfect case of reasoning, somewhat as follows: "If I can only succeed in passing this oilcloth I will be all right"?

Mr. McGillivray then proceeded to read his essay, "Illustrations of Animal Psychology," claiming that psychology was to-day the subject claiming attention from some of the greatest scientists of the day. Any person having to do with the rearing and caring of domestic animals, and who makes use of his opportunities, cannot fail to be impressed by the fact that many of their actions show reason to a certain extent. The following incident which occurred in our own streets a few days ago, was then related: A horse standing about fifteen feet from another wanted to approach his fellow, but was prevented by a weight which was attached to his halter; finally, however, he solved the problem by taking the strap in his mouth about a foot from the weight, which he carried to within reach of the horse with which he wished to communicate. A man in a similar position would have done exactly the same, and on his part it would be attributed to reason; why is it not reason in the horse?

Mr. Baker's essay was then listened to, "Reasons for Protection of Young," several very interesting features being emphasized in regard to this question.

The meeting then adjourned, not, however, before the president had complimented the essayists on the evident care shown in the preparation of their papers. He also, as in former years, suggested fields for investigation during vacation.

The society decided to write the secretary for the Prevention of Cruelty to Animals, expressing sympathy with them in their crusade against the sale of chameleons.

